# Identifying At-Risk Populations for Treatment Delays in Endometrioid Ovarian Carcinoma: A Nationally Representative Study

**DOI:** 10.3390/biomedicines13092065

**Published:** 2025-08-25

**Authors:** Isabella Zent, Kate Woods, Mitchell Taylor, Peter Silberstein, Megan Kalata

**Affiliations:** 1 School of Medicine, Creighton University, Omaha, NE 68178, USA; 2 Department of Internal Medicine, Advocate Illinois Masonic Medical Center, Chicago, IL 60657, USA; 3 Division of Hematology and Oncology, Department of Internal Medicine, Creighton University Medical Center, Omaha, NE 68124, USA; 4 Department of Obstetrics and Gynecology, Creighton University Medical Center, Omaha, NE 68124, USA

**Keywords:** health disparities, SEER database, ovarian cancer, endometrioid ovarian carcinoma, racial health disparities

## Abstract

**Background:** Endometrioid ovarian carcinoma is a subtype of epithelial ovarian carcinoma and is the second most common type of malignant ovarian neoplasm. Studies investigating delayed treatment of ovarian cancer have identified significant impacts on overall survival rates. This study utilizes the Surveillance, Epidemiology, and End Results (SEER) database to identify cases of endometrioid ovarian carcinomas and factors associated with delays in time to treatment (TTT) greater than one month. **Methods:** The SEER database was queried to identify females with biopsy-confirmed cases of ovarian endometrioid carcinoma from 2010 to 2015. Chi-square tests, two-sided Fisher’s exact tests, and multivariable binary logistic regressions were completed using SPSS version 29.0.2. Statistical significance was confirmed when *p* < 0.05. **Results:** A total of 11,235 relevant patients were identified within the SEER database. A majority were Non-Hispanic White (69.2%), aged 50–59 (30.1%), had an annual income of $75,000+ (58.9%), resided in urban communities (90.0%), and were diagnosed with AJCC stage 1 disease (62.0%). When investigating treatment, 94.9% of patients received treatment within 1 month of diagnosis, while 5.1% experienced a delay of over 1 month before starting treatment. Multivariable binary logistic regression analysis demonstrated that American Indian and Alaksa Native (AIAN) patients independently experienced a +376% increased likelihood of treatment delays exceeding 1 month (aOR 4.76; 95% CI 1.32–17.08; *p* = 0.017). Patients diagnosed at AJCC stage III (aOR 1.88; 95% CI: 1.22–2.91; *p* = 0.004) or stage IV (aOR: 4.50; 95% CI: 2.75–7.38; *p* < 0.001) additionally had +88% and +350% higher odds of treatment delays greater than 1 month, respectively. **Conclusions:** AIAN patients and those presenting with later stage disease for endometrioid ovarian carcinoma face significantly longer TTT, indicating disparities in timely care. Our findings demonstrate an urgent need for targeted interventions to address systemic barriers contributing to delayed treatment in these populations.

## 1. Introduction

Endometrioid ovarian carcinoma (EOVC) is a subtype of epithelial ovarian neoplasm that accounts for approximately 10–12% of ovarian epithelial malignancies [1]. It is the second most common form of epithelial ovarian carcinoma, following serous carcinoma, and has an increased risk of development in women with endometriosis [2,3]. There are important challenges in the diagnostic workup of this specific type of ovarian cancer, as EOVC represents a disproportionately large number of diagnostically difficult cases compared to its incidence among other gynecologic cancers [1]. Much of this diagnostic challenge is related to the ability of endometrioid carcinoma to present with a wide range of morphologies and immunophenotypes [1]. In comparison to other histological subtypes of ovarian cancer, EOVC has a better prognosis [2]. However, prognosis and disease-specific survival is affected by the many molecular factors that influence EOVC [2].

Standard treatment of ovarian cancer typically involves staging and primary debulking surgery, potentially followed by adjuvant chemotherapy depending on surgical staging [4]. Additional treatment, such as hormone therapy and maintenance therapy, may be employed depending on a variety of factors [4]. Standard treatment of EOVC follows similar guidelines [5]. Investigations into the treatment timeline of ovarian cancer have yielded important results demonstrating the need for timely intervention. Zhao et al. investigated 5-year overall survival (OS) and 5-year cancer-specific survival (CSS) between ovarian cancer patients receiving immediate treatment (within 1 month of diagnosis), intermediate delay to treatment (1–2 months after diagnosis), and long delay to treatment (3 or more months after diagnosis) [6]. This study found that both OS and CSS were significantly lower in patients who had a treatment delay greater than 1 month, with no statistical difference between the intermediate- and long-delay groups [6]. These findings support a recommended timeline of treatment within one month for ovarian cancer.

Despite the established survival benefits of early initiation of therapy for ovarian cancer, certain populations continue to experience disparities in treatment. African American women with ovarian cancer experience increased mortality compared to Non-Hispanic White women, likely related to unequal access to care, inadequate treatment regimens, and increased prevalence of comorbidities that can impact chemotherapy dosing [7,8,9]. The disparities in healthcare access experienced by African Americans are largely driven by the lower socioeconomic status and limited health insurance coverage that are typically observed in this population [8]. While these findings have been illustrated in multiple prior studies, there is a substantial gap in the literature regarding investigating factors associated with delayed treatment in ovarian carcinoma, particularly in the endometrioid subtype. The aim of this study, therefore, is to assess patient factors associated with delayed time to treatment (TTT) for EOVC in a representative cohort utilizing the Surveillance, Epidemiology, and End Results (SEER) database.

## 2. Materials and Methods

### 2.1. Data Source

Data regarding patient clinicopathological characteristics and TTT were collected using the SEER*Stat 8.4.5 software (National Cancer Institute/National Institute of Health, Bethesda, MD, USA) for the years from 2010 to 2015. The Surveillance, Epidemiology, and End Results (SEER) database is a population-based cancer registry that collects data on cancer incidence and survival data from 17 registries across the United States. Accounting for approximately 26.5% of the U.S. population, the SEER database provides detailed information on patient demographic, clinical, and survival characteristics [10]. Use of standardized data collection, broad geographic coverage, and long-term follow-up in the SEER database make it a valuable tool that is extensively used in epidemiological and clinical research to analyze trends in cancer incidence, treatment outcomes, and healthcare disparities. This study was considered exempt from Institutional Review Board (IRB) review, as the SEER database provides publicly accessible, de-identified data that adhere to ethical and privacy regulations.

### 2.2. Population Selection

The SEER-17 database was queried to identify biopsy-confirmed cases of ovarian endometrioid carcinoma that were diagnosed from 2010 to 2015. Cases were identified and selected using International Classification of Disease for Oncology 3rd edition (ICD-O-3) histology codes 8380/3-8383/3 and primary tumor location code C56.9. Patients with missing or unknown ICD-O-3 codes were excluded from the analysis.

### 2.3. Variables and Outcomes

Data on epidemiological variables—including age at diagnosis, race and ethnicity, annual income, and rural versus urban residence—were collected. Classification of rural and urban residency was determined using the SEER Rural–Urban Continuum codes, which were developed by the Economics Research Service of the U.S. Department of Agriculture [11]. Annual income, as defined by the SEER database, is based on median household income from the U.S. Census tract and county-level data and is not reported as individual or family income. Primary tumor location, tumor grade, and American Joint Committee on Cancer (AJCC) 7th edition disease stage were identified and recorded for disease characteristics. Disease staging for endometrioid ovarian carcinoma in our study was based on AJCC 7th edition, as data associated with more recent staging editions were not consistently available in the SEER database beyond our study period of 2010–2015. Receipts of surgical intervention, chemotherapy, and radiation therapy and time from diagnosis to treatment were collected. TTT was defined as the interval between diagnosis and initiation of definitive therapy. Patients with an unknown TTT were excluded from analysis.

### 2.4. Statistical Analysis

Data were transcribed and analyzed using SPSS for Mac, version 29.0.2 (IBM Corp., Armonk, NY, USA). Frequency tables were created to evaluate and describe baseline patient and disease characteristics. Pearson’s Chi-squared and two-sided Fischer’s exact tests were utilized to assess associations between categorical variables and TTT. To evaluate factors independently associated with treatment delays of >1 month, a multivariable binary logistic regression was performed adjusting for age at diagnosis, race and ethnicity, annual income, marital status, rural–urban residency, and AJCC 7th edition disease stage. The cutoff point of >1 month when evaluating TTT was decided based on prior literature demonstrating significantly worse survival outcomes in ovarian cancer patients with treatment delays beyond one month [6], thus supporting an optimal interval of less than one month when initiating treatment after diagnosis. To evaluate multicollinearity, a linear regression with TTT as the dependent variable was performed, including all variables utilized within the multivariable analysis. Variance inflation factors (VIFs) were <2 for all variables, assuring the model did not appear to be strongly impacted by multicollinearity. Statistical significance was considered when *p* < 0.05.

## 3. Results

A total of 11,235 patients with EOVC were identified from 2010 to 2015 using the SEER database (Table 1). The greatest number of patients were Non-Hispanic (NH) White (69.2%) followed by Hispanic (of any race) (14.0%), NH Asian or Pacific Islander (API) (11.2%), NH Black (5.1%), and NH American Indian and Alaska Native (AIAN) (0.5%). Regarding age distribution, the greatest number of patients were aged 50–59 (30.1%) followed by aged 40–49 (23.0%), 60–69 (20.4%), 70–79 (12.1%), <40 (9.0%), and 80+ (5.4%). The vast majority of patients resided in urban communities (90.0%), with a smaller proportion living in rural communities (10.0%). Additionally, more patients were married (55.4%) compared to those who were unmarried (44.6%). Income data demonstrated that most patients had an average annual income of greater than $75,000 (58.9%), whereas a smaller percentage earned less than $74,999 per year (41.1%). Concerning disease characteristics, the greatest number of patients had AJCC stage I disease (62.0%) at diagnosis, followed by stage II (16.9%), stage III (15.3%), and stage IV (5.8%). For treatment, surgical intervention was utilized in most patients, with 98.1% of patients undergoing surgery. Chemotherapy and radiation therapy were less frequently utilized, with 40.7% of patients receiving chemotherapy and 2.9% undergoing radiation therapy. Regarding TTT, the majority of patients had a reported TTT of ≤1 month (94.9%), whereas a smaller percentage had a TTT of >1 month (5.1%).

Comparing EOVC patients with TTT ≤ 1 month to those with TTT > 1 month, Chi-square analysis revealed several significant associations across several demographic and clinicopathological features (Table 2). There were significant associations between age at diagnosis and TTT (*p* < 0.001); younger patients aged <40 (9.3% vs. 7.6%), 40–49 (23.4% vs. 17.1%), and 50–59 (30.5% vs. 27.8%) were more likely to initiate treatment within one month of diagnosis, whereas ages 60–69 (22.8% vs. 20.2%), 70–79 (17.5% vs. 11.6%), and 80+ (7.2% vs. 5.0%) experienced increased likelihood of treatment delays >1 month. Patients who received treatment ≤1 month were also more likely to be NH White (68.6% vs. 62.2%) and NH API (11.5% vs. 10.3%), while a larger proportion of patients with TTT > 1 month were NH Black (8.7% vs. 5.0%), NH AIAN (0.7% vs. 0.5%), and Hispanic of any race (18.0% vs. 14.4%) (*p* < 0.001). TTT was also significantly associated with annual income (*p* = 0.028), where a greater percentage of individuals with a TTT ≤ 1 month were high-income patients earning $75,000+ annually (58.8%) compared to those earning <$74,999 (41.2%). Additionally, disease stage at diagnosis was another significant factor associated with TTT (*p* < 0.001). Patients undergoing treatment initiation ≤1 month were more likely to present with AJCC stage I (63.6% vs. 46.8%) and stage II (17.1% vs. 15.2%) disease at diagnosis, whereas individuals with treatment delays >1 month were more likely to present with stage III (20.9% vs. 14.6%) or stage IV (17.1% vs. 4.7%) disease. Patients with TTT > 1 month were also more likely to be unmarried (56.1% vs. 43.9%) (*p* < 0.001) and undergo chemotherapy (68.5% vs. 59.1%) (*p* < 0.001), whereas surgical treatment was more frequently utilized in patients with a TTT of ≤1 month (99.3% vs. 95.2%) (*p* < 0.001). There were no significant associations between TTT and rural–urban living (*p* = 0.757) or receipt of radiation therapy (*p* = 0.232).

Multivariable analysis, while adjusting for age at diagnosis, race and ethnicity, annual income, marital status, rural–urban residency, and AJCC disease stage, was utilized to further identify demographic and clinicopathologic features independently associated with a TTT >1 month (Table 3). Compared to NH White patients, NH AIAN patients experienced a +376% increased likelihood of TTT >1 month (adjusted odds ratio [aOR]: 4.76; 95% confidence interval [CI: 1.32–17.08]; *p* = 0.017). Additionally, patients diagnosed at AJCC stage III had significantly higher odds of experiencing a TTT >1 month (aOR 1.88; 95% CI: 1.22–2.91; *p* = 0.004), as did those with stage IV disease (aOR: 4.50; 95% CI: 2.75–7.38; *p* < 0.001). 

## 4. Discussion

Endometrioid ovarian carcinoma (EOVC) is the second most common subtype of epithelial ovarian cancer that frequently develops in females with endometriosis [2,3]. Outcomes in ovarian carcinoma are significantly impacted by TTT initiation, with delays greater than one month demonstrating reduced overall and cancer-specific survival [6]. This study utilized the SEER database to analyze factors related to disparities in TTT of EOVC. We found that 94.9% of patients were able to receive treatment within one month of diagnosis, while only 5.1% experienced a treatment delay. Our study identified that disease stage and race and ethnicity are significantly associated with delayed TTT.

According to current literature, EOVC typically appears at earlier stages of disease than other types of ovarian cancer [2]. This is reflected in our results as 62.0% of patients presented with AJCC stage I disease at diagnosis, 16.9% with AJCC stage II, 15.3% with AJCC stage III, and only 5.8% with AJCC stage IV disease. Interestingly, we observed a significant association between disease stage and treatment delays, with our multivariable analysis demonstrating that disease stage at diagnosis is independently associated with TTT. AJCC stage III and IV were associated with +88% and +350% higher odds, respectively, of delayed treatment (aOR 1.88; 95% CI: 1.22–2.91; *p* = 0.004; aOR: 4.50; 95% CI: 2.75–7.38; *p* < 0.001) when compared to patients diagnosed with AJCC stage I disease. This finding may be related to increased complexity of the cases requiring more extensive diagnostic workup and additional time to thoroughly plan and execute interventions. Patients diagnosed at a later stage may also face sociodemographic barriers that limit access to routine medical care, specialty-specific and subspecialty-specific care, and coordination of extensive surgical interventions [12,13,14].

In addition to disease stage at presentation, racial and ethnic background were shown to be significantly associated with TTT initiation. Multivariable analysis revealed that AIAN individuals experienced a +376% higher chance of treatment delays greater than 1 month, even after adjusting for potential confounding variables (aOR 4.76; 95% CI: 1.32–17.08; *p* = 0.017). This disparity in TTT may reflect long-standing barriers in access to obstetric and gynecologic care and frequently diminished outcomes among the AIAN population, which have many potential contributing factors [15,16,17,18,19,20,21]. Much of the literature surrounding the health of the United States AIAN population, both general and specific to the OB/GYN specialty, cites general longstanding mistrust of the healthcare system [15,16]. A qualitative study exploring opinions on traditional and Western healthcare among members of a Gulf Coast tribe found themes of preference towards traditional medicine, avoidance of Western medical practices, and negative interactions with providers [17]. It is important to recognize that the experiences and opinions of tribal members may vary widely across community, language, cultural practices, geographic space, and political and sociodemographic history [16]. However, this study by Reese et al. provides insight into another potential barrier for some AIAN patients when seeking treatment from Western medical facilities. Furthermore, AIAN patients also continue to experience a disproportionate burden of chronic disease, low rate in clinical trial enrollment, underuse of preventative care, and mortality related to cancer [16,18]. Many of these pervasive issues likely affect all areas of care and access to timely treatment, including OB/GYN care. Within this specialty, AIAN patients experience disparities including but not limited to a cervical cancer mortality rate twice that of the general U.S. population, a higher likelihood of infection with high-risk HPV strains not protected against the nonvalent vaccine, poorer resolution of endometriosis-related pain following treatment, and increased odds of surgical complications during endometriosis treatment [15,19,20]. Barriers that AIAN patients may face in obtaining gynecologic oncology care include the need to be referred to providers outside the Indian Health Service (IHS), lack of funding for the IHS Purchased and Referred Care program, geographical and transportation barriers in traveling extensive distances to appointments, and lack of sufficient coverage through insurance [16,21]. Interestingly, our study did not demonstrate a statistically significant difference in TTT between urban and rural populations (*p* = 0.757). According to the IHS, approximately 87% of the AIAN population lives in urban areas while 13% live on reservations or tribal land, suggesting that AIAN disparities may be due to factors beyond area of residence [22].

The disparities highlighted in our study underscore the need for direct and targeted interventions to decrease the burden of treatment delay on specific populations. At the provider level, patient education and mutual dialogue should be implemented to formulate a treatment plan that is in the best interest of the patient and takes into consideration their individual barriers to care. Effectively engaging patients in their care and in the medical decision-making process is crucial for improving health literacy and compliance, with the additional benefits of reducing care costs and improving patient satisfaction [23]. Additionally, this form of patient-centered care allows health professionals to direct referrals to other services that may alleviate the burden of social determinants of health on cancer care, such as social work, community outreach programs, or other patient educators [24,25]. On a larger scale, awareness of these disparities may allow healthcare systems to increase targeted outreach for at-risk communities. While National Cancer Institute-designated cancer centers and the IHS both employ community outreach and engagement programs [25,26], improving communication and collaboration between these two entities may improve referral pathways, transportation options, cultural awareness, and overall ability of AIAN patients to obtain more timely cancer treatment. Our findings suggest that more targeted interventions may be required to connect specific populations with resources, education, referrals, and treatment options to alleviate disparities in accessing cancer care for patients with EOVC.

Although the SEER database provides access to valuable national data, there are inherent limitations to this investigation due to its retrospective nature, which limits causal inferences. Additionally, there is a lack of patient data incorporating the most recent AJCC 8th edition staging guidelines in the SEER database; thus, the 7th edition was utilized, which encompasses the years 2010–2015. As such, more recent advances in the field of OB/GYN oncologic care may not be fully reflected in our study findings. There is also little published data investigating the consequences of delayed treatment of endometrioid ovarian carcinoma specifically; therefore, limited conclusions may be drawn about morbidity and mortality consequences of delayed TTT from this study. Additionally, the SEER database lacks data on certain potentially confounding variables, such as comorbidity burden, performance status, and detailed treatment planning factors, which may also influence treatment delays. While disease-specific survival analyses were conducted to minimize the influence of unrelated causes of death—including from patient comorbidities—on our measured outcomes, some of these factors could play a role in EOVC patients’ ability to obtain care in a timely manner and, thus, are limitations of our study. Finally, due to variations in population sizes between race/ethnicity categories, statistical significance may not be directly representative of clinical significance.

## 5. Conclusions

This study demonstrates a significant delay in the treatment timeline of endometrioid ovarian carcinoma for AIAN patients and those presenting in later stage disease. There are many potential factors contributing to the delayed treatment of patient subgroups. The consequences of this delayed treatment timeline should be further investigated to understand the scope of clinical consequence to patients. Additionally, this identified delay may indicate a more pervasive issue in the treatment of gynecologic cancer patients across ovarian cancer subtypes. Reasons behind these delays in treatment should be investigated and followed over time to ensure equitable cancer care for AIAN patients.

## Figures and Tables

**Table 1:** Clinicopathologic Features of Ovarian Endometrioid Carcinoma Cohort.

Total *n* = 11,235	Frequency (N)	Percentage (%)
**Age at diagnosis (years)**		
<40	1014	9.0
40–49	2580	23.0
50–59	3386	30.1
60–69	2287	20.4
70–79	1357	12.1
80+	611	5.4
**Race and ethnicity**		
NH White	7740	69.2
NH Black	566	5.1
NH API	1252	11.2
NH AIAN	59	0.5
Hispanic (any race)	1572	14.0
**Annual income**		
<$74,999	4621	41.1
$75,000+	6613	58.9
**Marital status**		
Married	5994	55.4
Not married	4834	44.6
**Rural-urban living**		
Urban	10,104	90.0
Rural	1122	10.0
**AJCC stage**		
Stage I	1833	62.0
Stage II	500	16.9
Stage III	451	15.3
Stage IV	171	5.8
**Surgery**	11,016	98.1
**Chemotherapy**	4573	40.7
**Radiation therapy**	325	2.9
**Time to treatment**		
≤1 month	10,014	94.9
>1 month	543	5.1

AIAN, American Indian and Alaska Native; API, Asian or Pacific Islander; NH, Non-Hispanic.

**Table 2:** Comparison of Clinicopathologic Features by TTT for Endometrioid Carcinoma Cohort.

	TTT ≤ 1 Month (*n* = 10,014)	TTT > 1 Month (*n* = 543)	*p*-Value
**Age at diagnosis (years)**			**<0.001** ^Δ^
<40	934 (9.3%)	41 (7.6%)	
40–49	2340 (23.4%)	93 (17.1%)	
50–59	3058 (30.5%)	151 (27.8%)	
60–69	2018 (20.2%)	124 (22.8%)	
70–79	1164 (11.6%)	95 (17.5%)	
80+	500 (5.0%)	39 (7.2%)	
**Race and ethnicity**			**<0.001** ^Δ^
NH White	6843 (68.6%)	338 (62.2%)	
NH Black	501 (5.0%)	47 (8.7%)	
NH API	1144 (11.5%)	56 (10.3%)	
NH AIAN	51 (0.5%)	4 (0.7%)	
Hispanic (any race)	1431 (14.4%)	98 (18.0%)	
**Annual income**			**0.028** °
<$74,999	4125 (41.2%)	250 (46.0%)	
$75,000+	5888 (58.8%)	293 (54.0%)	
**Marital status**			**<0.001** °
Married	5398 (55.9%)	232 (43.9%)	
Not married	4251 (44.1%)	297 (56.1%)	
**Rural-urban living**			0.757 °
Urban	9054 (90.5%)	494 (91.0%)	
Rural	951 (9.5%)	49 (9.0%)	
**AJCC stage**			**<0.001** ^Δ^
Stage I	1749 (63.6%)	74 (46.8%)	
Stage II	471 (17.1%)	24 (15.2%)	
Stage III	400 (14.6%)	33 (20.9%)	
Stage IV	128 (4.7%)	27 (17.1%)	
Surgery *	9939 (99.3%)	517 (95.2%)	**<0.001** °
Chemotherapy *	5915 (59.1%)	372 (68.5%)	**<0.001** °
Radiation therapy *	281 (2.8%)	20 (3.7%)	0.232 °

Significant *p*-values (<0.05) are in bold. AIAN, American Indian and Alaska Native; API, Asian or Pacific Islander; NH, Non-Hispanic. ^Δ^ Test statistic calculated using Chi-square test. ° Test statistics calculated using two-sided Fisher’s exact test. * Compared to not receiving this treatment.

**Table 3:** Multivariable Logistic Regression Identifying Factors Associated with Treatment Delays >1 Month in Ovarian Endometrioid Carcinoma Patients.

	aOR ‡	95% CI	*p*-Value
**Age at diagnosis (years)**			
<40	Reference		
40–49	0.78	0.40–1.55	0.485
50–59	0.76	0.40–1.48	0.422
60–69	1.05	0.54–2.06	0.885
70–79	1.02	0.48–2.17	0.954
80+	1.63	0.74–3.61	0.228
**Race and ethnicity**			
NH White	Reference		
NH Black	1.02	0.49–2.12	0.963
NH API	1.16	0.69–1.97	0.576
NH AIAN	4.76	1.32–17.08	**0.017**
Hispanic (any race)	1.17	0.74–1.84	0.496
**Annual income**			
<$74,999	Reference		
$75,000+	0.82	0.58–1.16	0.264
**Marital status**			
Married	Reference		
Not married	1.19	0.85–1.67	0.322
**Rural-urban living**			
Urban	Reference		
Rural	0.82	0.44–1.51	0.519
**AJCC Stage**			
Stage I	Reference		
Stage II	1.27	0.79–2.05	0.325
Stage III	1.88	1.22–2.91	**0.004**
Stage IV	4.50	2.75–7.38	**<0.001**

Significant *p*-values (<0.05) are in bold. AIAN, American Indian and Alaska Native; API, Asian or Pacific Islander; NH, Non-Hispanic. This model was adjusted for the following categorical variables: age at diagnosis, race and ethnicity, annual income, marital status, rural–urban living, and AJCC 7th edition disease stage. To evaluate multicollinearity, a linear regression with TTT as the dependent variable was performed, including all variables used in the multivariable analysis. Variance inflation factors (VIFs) were <2 for all variables, so the model did not appear to be strongly impacted by multicollinearity. ‡ Adjusted odds ratio refers to odds of having a TTT of >1 month after adjusting for important covariates.

## Data Availability

The data that support the findings of this study are publicly available from the National Cancer Institute’s Surveillance, Epidemiology, and End Results (SEER) Program. The data used in this study were obtained from the SEER*Stat database (https://seer.cancer.gov/seerstat/ accessed on 29 July 2025) following approval of a SEER data use agreement, and all analyses were conducted in accordance with SEER’s guidelines for data use and confidentiality.

## Abbreviations

The following abbreviations are used in this manuscript:

AIAN	American Indian and Alaska Native
API	Asian or Pacific Islander
EOVC	Endometrioid ovarian carcinoma
NH	Non-Hispanic
SEER	Surveillance Epidemiology and End Results
TTT	Time to treatment

## References

[1] Talia K.L., Mccluggage W.G. (2023). The diverse morphology and immunophenotype of ovarian endometrioid carcinomas. Pathology.

[2] Chen S., Li Y., Qian L., Deng S., Liu L., Xiao W., Zhou Y. (2021). A review of the clinical characteristics and novel molecular subtypes of endometrioid ovarian cancer. Front Oncol..

[3] Steinbuch S.C., Lüß A., Eltrop S., Götte M., Kiesel L. (2024). Endometriosis-associated ovarian cancer: From molecular pathologies to clinical relevance. Int. J. Mol. Sci..

[4] Arora T., Mullangi S., Vadekekut E.S., Mandihar R.L. Epithelial Ovarian Cancer. StatPearls Web Site. https://www.ncbi.nlm.nih.gov/books/NBK567760/.

[5] (2025). NCCN Clinical Practice Guidelines in Oncology for Ovarian Cancer/Fallopian Tube Cancer/Primary Peritoneal Cancer, Version 2. https://www.nccn.org/guidelines/guidelines-detail?category=1&id=1453.

[6] Zhao J., Chen R., Zhang Y., Wang Y., Zhu H. (2024). Impact of treatment delay on the prognosis of patients with ovarian cancer: A population-based study using the surveillance, epidemiology, and end results database. J. Cancer.

[7] Chornokur G., Amankwah E.K., Schildkraut J.M., Phelan C.M. (2014). Global ovarian cancer health disparities. Gynecol. Oncol..

[8] Terplan M., Smith E.J., Temkin S.M. (2009). Race in ovarian cancer treatment and survival: A systematic review with meta-analysis. Cancer Causes Control.

[9] Bandera E.V., Lee V.S., Rodriguez-Rodriguez L., Powell C.B., Kushi L.H. (2015). Impact of chemotherapy dosing on ovarian cancer survival according to body mass index. JAMA Oncol..

[10] SEERRegistries—About S.E.E.R. National Cancer Institute—Surveillance, Epidemiology, and End Results Program. https://seer.cancer.gov/registries/.

[11] US Department of Agriculture, Economic Research Service (2024). Rural-Urban Continuum Codes. https://www.ers.usda.gov/data-products/rural-urban-continuum-codes.

[12] Præstegaard C., Kjaer S.K., Nielsen T.S., Jensen S.M., Webb P.M., Nagle C.M., Høgdall E., Risch H.A., Rossing M.A., Doherty J.A. (2017). The association between socioeconomic status and tumour stage at diagnosis of ovarian cancer: A pooled analysis of 18 case-control studies. Cancer Epidemiol..

[13] Singh S., Sridhar P. (2021). A narrative review of sociodemographic risk and disparities in screening, diagnosis, treatment, and outcomes of the most common extrathoracic malignancies in the United States. J. Thorac. Dis..

[14] Clegg L.X., Reichman M.E., Miller B.A., Hankey B.F., Singh G.K., Lin Y.D., Goodman M.T., Lynch C.F., Schwartz S.M., Chen V.W. (2010). Impact of socioeconomic status on cancer incidence and stage at diagnosis: Selected findings from the surveillance, epidemiology, and end results: National longitudinal mortality study. Cancer Causes Control.

[15] Buck Disilvestro J., Ulmer K.K., Hedges M., Kardonsky K., Bruegl A.S. (2024). Cervical cancer: Preventable deaths among American Indian/Alaska Native communities. Obstet. Gynecol. Clin. N. Am..

[16] Warne D., Baker T., Burson M., Kelliher A., Buffalo M., Baines J., Whalen J., Archambault M., Jinnett K., Mohan S.V. (2025). Barriers and unmet needs related to healthcare for American Indian and Alaska Native communities: Improving access to specialty care and clinical trials. Front. Health Serv..

[17] Reese S.E., Dang A., Liddell J.L. (2024). ‘We’d just patch ourselves up’: Preference for holistic approaches to healthcare and traditional medicine among members of a state-recognized tribe. J. Holist Nurs..

[18] Bruegl A.S., Emerson J., Tirumala K. (2024). Persistent disparities of cervical cancer among American Indians/Alaska Natives: Are we maximizing prevention tools?. Gynecol. Oncol..

[19] Spagnolia A., Beal J.R., Sahmoun A.E. (2020). Differences in clinical management and outcomes of American Indian and white women diagnosed with endometriosis. J. Fam. Reprod. Health.

[20] Orlando M.S., Russo M.A.L., Richards E.G., King C.R., Park A.J., Bradley L.D., Chapman G.C. (2022). Racial and ethnic disparities in surgical care for endometriosis across the United States. Am. J. Obstet. Gynecol..

[21] Akapo A.O., Schultz C., Coelho D., Muffly T.M. (2023). American Indian and Alaskan Native access to obstetrics and gynecology subspecialists: Findings from a national mystery caller study in the United States. Cureus.

[22] Indian Health Service Office of Urban Indian Health Programs. Indian Health Service Web Site. https://www.ihs.gov/urban/aboutus/about-urban-indian-organizations/.

[23] Krist A.H., Tong S.T., Aycock R.A., Longo D.R. (2017). Engaging Patients in Decision-Making and Behavior Change to Promote Prevention. Stud. Health Technol. Inform..

[24] Zebrack B., Zhang A., Ghazal L.V., Francis-Levin N., Brandon R.E. (2025). The Essential Nature of Social Work in Cancer Control. Cancer Control.

[25] Ramanadhan S., Daly J., Lee R.M., Mallick K., Augenbraun S.L., Emmons K.M. (2024). Maximizing the impact of community outreach and engagement at US cancer centers. JNCI Cancer Spectr..

[26] Community Health Representative (CHR) Program “About Us”. Indian Health Service. https://www.ihs.gov/chr/aboutus/.

